# COVID-19 Conceptual Modeling: Single-Center Cross-Sectional Study

**DOI:** 10.2196/41376

**Published:** 2023-07-11

**Authors:** Mawahib Abuauf, Enaam Hassan Raboe, Muneera Alshareef, Nada Rabie, Roaa Zailaie, Abdullah Alharbi, Walaa Felemban, Ibrahim Alnasser, Hanin Shalaby

**Affiliations:** 1 Pediatric Department King Fahad Armed Forces Hospital Jeddah Saudi Arabia; 2 Department of Surgery Pediatric Surgery Division King Fahad Armed Forces Hospital Jeddah Saudi Arabia; 3 Adult Endocrinology Services King Fahad Armed Forces Hospital Jeddah Saudi Arabia; 4 Department of Medicine King Fahad Armed Forces Hospital Jeddah Saudi Arabia; 5 Medical Laboratory Services – Histopathology King Fahad Armed Forces Hospital Jeddah Saudi Arabia; 6 Radiology Services King Fahad Armed Forces Hospital Jeddah Saudi Arabia; 7 Clinical Research and Data Management King Fahad Armed Forces Hospital Research Center Jeddah Saudi Arabia

**Keywords:** conceptual model, conceptual framework, modeling, Saudi Arabia, COVID-19, SARS-CoV-2, mortality, morbidity, CURB-65, socioeconomic, health care, hospital database, electronic record, medical decision-making, hospital admission, hospitalization, cross-sectional, death, intensive care

## Abstract

**Background:**

Conceptual models are abstract representations of the real world. They are used to refine medical and nonmedical health care scopes of service. During the COVID-19 pandemic, numerous analytic predictive models were generated aiming to evaluate the impact of implemented policies on mitigating the spread of the virus. The models also aimed to examine the psychosocial factors that might govern the general population’s adherence to these policies and to identify factors that could affect COVID-19 vaccine uptake and allocation. The outcomes of these analytic models helped set priorities when vaccines were available and predicted readiness to resume non–COVID-19 health care services.

**Objective:**

The objective of our research was to implement a descriptive-analytical conceptual model that analyzes the data of all COVID-19–positive cases admitted to our hospital from March 1 to May 31, 2020, the initial wave of the pandemic, the time interval during which local policies and clinical guidelines were constantly updated to mitigate the local effects of COVID-19, minimize mortality, reduce intensive care unit (ICU) admission, and ensure the safety of health care providers. The primary outcome of interest was to identify factors that might affect mortality and ICU admission rates and the impact of the implemented policy on COVID-19 positivity among health care providers. The secondary outcome of interest was to evaluate the sensitivity of the COVID-19 visual score, implemented by the Saudi Arabia Ministry of Health for COVID-19 risk assessment, and CURB-65 (confusion, urea, respiratory rate, blood pressure, and age >65 years) scores in predicting ICU admission or mortality among the study population.

**Methods:**

This was a cross-sectional study. The relevant attributes were constructed based on research findings from the first wave of the pandemic and were electronically retrieved from the hospital database. Analysis of the conceptual model was based on the International Society for Pharmacoeconomics and Outcomes Research guidelines and the Society for Medical Decision-Making.

**Results:**

A total of 275 individuals tested positive for COVID-19 within the study design interval. The conceptualization model revealed a low-risk population based on the following attributes: a mean age of 42 (SD 19.2) years; 19% (51/275) of the study population being older adults ≥60 years of age; 80% (220/275) having a CURB-65 score <4; 53% (147/275) having no comorbidities; 5% (13/275) having extreme obesity; and 20% (55/275) having a significant hematological abnormality. The overall rate of ICU admission for the study population was 5% (13/275), and the overall mortality rate was 1.5% (4/275). The multivariate correlation analysis revealed that a high-selectivity approach was adopted, resulting in patients with complex medical problems not being sent to MOH isolation facilities. Furthermore, 5% of health care providers tested positive for COVID-19, none of whom were health care providers allocated to the COVID-19 screening areas, indicating the effectiveness of the policy implemented to ensure the safety of health care providers.

**Conclusions:**

Based on the conceptual model outcome, the selectivity applied in retaining high-risk populations within the hospital might have contributed to the observed low mortality rate, without increasing the risk to attending health care providers.

## Introduction

The outbreak of SARS-CoV-2 was identified in December 2019 by health authorities in Wuhan, China, based on a cluster of symptoms suggestive of atypical pneumonia that did not resolve with 3-5 days of antibiotics [1].

The probability of a new zoonosis was considered, and the clinical presentation was similar to that of the SARS-CoV-1 that broke out in 2003, connecting the coronavirus to severe respiratory distress. The main feature of the new outbreak was high contagiousness suggestive of human-to-human transmission. On March 11, 2020, the World Health Organization declared SARS-CoV-2 (COVID-19) a pandemic.

An extraordinarily rapid and effective scientific response followed this announcement; the causative pathogen was isolated, the virus genome sequence was identified, diagnostic tests were developed, and studies were conducted to evaluate the environmental survival of COVID-19 [2]. The virus was proposed to use angiotensin-converting enzyme receptors to infect humans. However, the role of angiotensin-converting enzyme 2 or angiotensin II receptor blocker therapy in disease severity was unclear, and no conclusion could be drawn from the initial published reports [3]. Notably, the information gained from these studies had a variable impact on disease progression in different countries. Therefore, modeling was reintroduced as a tool to evaluate the medical and nonmedical effects of COVID-19. Modeling is defined as an abstract representation of the real world linked to learning theory. Modeling is case sensitive, as it is affected by the attributes included in the model and the questions to be answered; thus, different models are developed for different purposes. Gofftried Leibniz introduced the concept of mathematical modeling of health problems in the 17th century, and William Farr was the first to employ the concept during the cholera epidemic in 1866. Furthermore, Kermack and McKendric (1927) introduced the susceptible, infected, and recovered epidemiology model, which predicts disease progression in a population of interest [4]. However, the limitation of this model is that it assumes compartmental homogeneity [4]. Various prediction models have been implemented in medical care. Cichoz et al [5], for example, summarized the different models developed to predict the short- and long-term complications of diabetes to optimize medical care.

During the COVID-19 pandemic, several models were generated. Azizur-Rahman [6] summarized the models used in China, the United Kingdom, New South Wales, and Australia. These models evaluated the impacts of nonpharmacological interventions (NPIs), such as social distancing, the use of facial masks, bans on international travel, as well as local and regional segregation on pandemic propagation; the outcomes indicated that these interventions had an impact on the progression of the pandemic; thus, the systematic, controlled elevation of lockdowns to delay and ameliorate the pandemic’s second peak was proposed. Additionally, Fox et al’s [7] mathematical model evaluated the impact of NPIs on COVID-19 hospitalization and ICU admission and the time to the second peak in New South Wales, Australia. The model revealed that NPIs reduced hospitalization and intensive care unit (ICU) admissions by 50% and delayed the second peak by 3 months. These findings enforced the policies implemented and medical guidelines. In addition, Maclntyre et al’s [8] model predicted the impact of vaccination on COVID-19 containment, indicating that the vaccination of health care workers is needed to ensure their resilience, while the vaccination of older individuals would decrease mortality and ICU admissions. This helped in setting vaccination priorities once vaccines were available.

Conceptual modeling is a subset of data modeling. It has been defined as a “representation of a system that uses concepts and ideas to form said representation” [9]. A conceptual model thus is a system of concepts, assumptions, expectations, beliefs, and theories that support and inform a research framework. Conceptual models are an abstract representation of the real world and are used across many fields, such as the sciences, socioeconomics, and software development [10]. Conceptual models can be reported graphically as data visualizations or as a narrative text representing key factors, concepts, and attributes to address the presumed relationships between them. Gray and Sockolow [11] reviewed conceptual modeling and its relevance to health care, suggesting that conceptual models might bridge the gap between health care information and technology. Brady et al’s [12] conceptual model identified a number of nonmedical variables that might be associated with adult lower urinary tract symptoms. These findings need further verification via classical clinical research. Gustafson et al [13] evaluated the managerial role of conceptual modeling by developing a Bayesian model that predicts health care organization success, and Hervatis et al [14] implemented a conceptual model to guide postgraduate medical education curriculum development. Finally, Rocco and Plakhotnik’s [15] review highlighted the similarities and differences between the theoretical and conceptual frameworks; the main distinction was that a theory might not be a driving factor in a conceptual framework.

Considering the medical and nonmedical role of the conceptual model, the objective of our research was to implement a conceptual model that analyzes the data of all COVID-19–positive cases admitted to our hospital from March 1 to May 31, 2020, the initial wave of the pandemic, the time interval during which local policies and clinical guidelines were constantly updated to mitigate the local effects of COVID-19, minimize mortality and ICU admission, and ensure the safety of health care providers. The primary outcome of interest was to identify factors that might affect mortality and ICU admission as well as the impact of the policy implemented regarding COVID-19 positivity among health care providers. The secondary outcome of interest was to evaluate the sensitivity of the COVID-19 visual score implemented by the Saudi Arabia Ministry of Health (MOH) for COVID-19 risk assessment as well as CURB-65 (confusion, urea, respiratory rate, blood pressure, and 65 years of age or older) scores in predicting ICU admissions or mortality among the study population. The CURB-65 is a validated scoring system developed to predict the need for ICU admission and mortality [16] among adults diagnosed with community-acquired pneumonia, based on confusion, urea, respiratory rate, blood pressure, and age in years.

## Methods

### Ethical Considerations

This was a cross-sectional study conducted from March 1 to May 31, 2020, at King Fahad Armed Hospital, Jeddah. As researchers, we questioned the feasibility of obtaining informed consent. Considering the pandemic time interval—the timing of the study’s conduct—obtaining an informed consent was not feasible. Thus, a proposal was submitted for ethical board review, evaluation, and approval, with the aim of waiving the requirement for informed consent and based on the expectation that no harm would occur to the patient [17-19]. With the ethical review board approval, we were permitted access to patients’ information according to hospital policies and regulations. As we submitted the research for publication, we revisited the issue of waiving informed consent and discussed the factor that led to the initial waiver, and thus, we obtained a second approval from the ethical review board chairperson to waive the requirement for informed consent.

### Attributes Extraction and Patients’ Confidentiality

**Figure 1:**
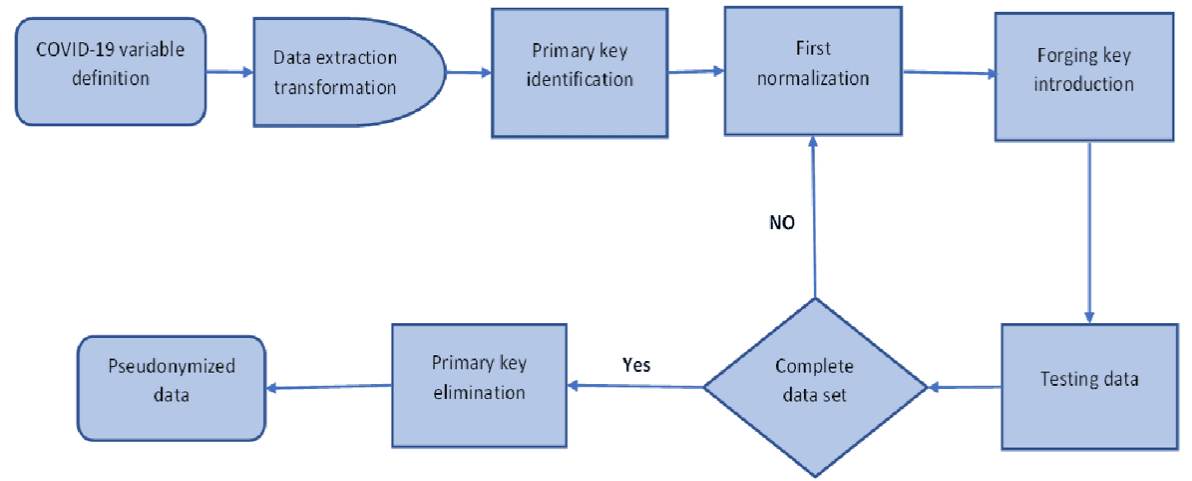
COVID-19 variable extraction pseudonymization process. Primary keys are unique variables that identify each participant’s data set. Forging keys are unique variables that are introduced to each data set to link a set of variables to their original data set.

The study population comprised patients who tested positive for COVID-19 and were admitted to our hospital within the defined time interval. The conceptual model was implemented following the International Society for Pharmacoeconomics and Outcomes Research guidelines and the Society for Medical Decision-Making for model transparency and validation [20]. Furthermore, the attributes used to construct the conceptual model were based on the risk factors reported in the literature associated with increased mortality or ICU admission among COVID-19–positive cases; these risk factors were electronically retrieved from the hospital database. The following variables were used to build the model: age; sex; BMI; hospital screening site of suspected cases; history of exposure to COVID-19–positive cases; presenting symptoms; COVID-19 visual scores; the need for intensive care; the number of antibiotics given; COVID-19–targeted medication; the need for steroid rescue therapy; medical-surgical comorbidities; history of chronic steroid use; the use of angiotensin-converting enzyme or angiotensin II receptor blocker medication; laboratory results within the first 3 days of presentation, including complete blood count, coagulation assay, blood urea nitrogen, serum albumin, C-reactive protein, and natriuretic brain peptide; blood cultures taken during hospitalization; hemoglobin A_1c_ (HbA_1c_) within 3 months of presentation; the occupation of health care providers who tested positive for COVID-19 during the study period; disease outcome; the length of hospital stay; and transfer site. These variables were extracted into Microsoft Excel sheets, and a series of transformation normalizations were carried out by a nominated hospital data scientist under the guidance of the corresponding author. Patients’ identifying variables were replaced with a unique number used to build the pseudonymized data set for subsequent analysis. These steps are presented in Figure 1. Access to the original data set was limited to the data scientist and correspondent author to ensure patient confidentiality.

### Statistical Methods

Our conceptual model was a single application with no mathematical equations. Yet the potential association between the input and output variables was verified using standard statistical calculations [21] to assess internal validity. The following statistical calculations were applied to the results obtained via the model. In the univariate analyses, demographic, clinical, and laboratory population characteristics by transformation status were examined using Fisher exact test, a chi-square test, and a 2-tailed *t* test, as appropriate. The assumptions of normality and homogeneity of variance were also assessed. Additionally, the time-to-transfer rates were estimated using the Kaplan-Meier product-limit method, and the differences between the curves for the transferred versus nontransferred patients were assessed using the log-rank test for patients transferred for a specific cause. In the multivariate analyses, a general linear model was used to examine predictors for the length of hospital stay. The assumptions of normality and homogeneity of variance were also assessed, and no violation of the assumptions was observed. For the second analysis, we used the exact binary multiple logistic regression model to examine the association between transfer status and all other covariates. Exact logistic regression accounted for fewer than 5 subjects per cell and 0 cells. The linear relationship between the logit of the outcome and each predictor variable was then assessed. All statistical tests were 2-sided, and findings were considered statistically significant at *P*<.05. All analyses were performed using SAS statistical software (version 9.4; SAS Institute Inc). Both cross-validity and external validity were also applied as indicated.

## Results

### Patient Characteristics

A total of 275 individuals tested positive for COVID-19 were identified, and their demographic characteristics are summarized in Table 1.

**Table 1:** Demographic characteristics of the study population.

Variable		Main result
Age (years), mean (SD), minimum-maximum		42 (19.2), 1-85
**Age distribution, n (%)**		
	Pediatric	18 (6.5)
	Adolescent	13 (4.7)
	Adult	193 (70.3)
	Older adult	51 (18.5)
**Gender, n (%)**		
	Male	166 (60)
	Female	109 (40)
Contact with COVID-19–positive cases (yes), n (%)		84 (30)
**Obesity, n (%)**		
	Overall frequency	78 (28)
	Adults with extreme obesity	13 (5)
**Comorbidities, n (%)**		
	None	147 (53)
	Present	128 (47)
Intensive care unit admission (yes), n (%)		14 (5)
Chronic steroid therapy (yes), n (%)		12 (4)
Angiotensin-converting enzyme inhibitors (yes), n (%)		25 (10)
**CURB-65^a^ score, n (%)**		
	Low risk	220 (80)
	High risk	55 (20)
**Outcome, n (%)**		
	Deceased	4 (1.5)
	Alive	271 (98.5)
Health care provider tested positive for COVID-19		13 (4.7)

^a^CURB-65: confusion, urea, respiratory rate, blood pressure, and age >65 years.

### The Relevance of Demographic Characteristics

The demographic attributes of the patients suggested of a low-risk population, with a mean age of 42 (SD 19.2) years; 19% (51/275) of the study population were older adults ≥60 years of age. Additionally, 33% (93/275) of the study population had obesity; 5% (13/275) of them had extreme obesity. Obesity was estimated based on the World Health Organization’s recommendations for different age categories. Furthermore, 53% (147/275) of the study population had no comorbidities; 80% (220/275) had low CURB-65 scores; and 19% (54/275) were diabetic, of whom 57% (31/54) had an HbA_1c_ level ≥7.5, reflecting poor control. The study population’s laboratory results revealed that 20% (55/275) had a platelet count ≤100,000, and 22% (60/275) had urea levels >7mmol/L. All the laboratory results are presented in Table 2.

**Table 2:** Laboratory results.

Laboratory category		Measurement
White blood cell count, median (Q1-Q3)		4.8 (3.7-6.5)
Neutrophil count, median (Q1-Q3)		2.5 (1.7-3.7)
Lymphocyte count, median (Q1-Q3)		1.5 (1.1-2.02)
Platelet count^a^, median (Q1-Q3)		226 (181-179)
International normalized ratio, median (Q1-Q3)		.99 (.99-1.05)
Blood urea nitrogen count^b^, median (Q1-Q3)		3.7 (2.9-4.8)
Serum albumin (g/L), median (Q1-Q3)		41 (37-43)
C-reactive protein count^c^, median (Q1-Q3)		10 (4.2-28.4)
**HbA_1c_^d^ (%; N=54), n (%)**		
	≤6.4	13 (24)
	6.5-6.9	3 (6)
	7-7.4	7 (13)
	≥7.5	31 (57)

^a^Of the study population, 20% (55/275) had platelet levels ≤100,000.

^b^Of the study population, 88% (242/275) had blood urea nitrogen levels <7mmol/L.

^c^The C-reactive protein cutoff reference value in our lab was 6.

^d^A total of 54 patients in the study population had hemoglobin A_1c_ (HbA_1c_) measured, of whom 52 were diabetic; 57% (31/54) of the screened patients had HbA_1c_ levels >7.4%, reflecting poor glycemic control. The HbA_1c_ classification was based on Ministry of Health guidelines.

### The Implemented Hospital Policy’s Impact on COVID-19 Positivity Among Health Care Providers

**Figure 2:**
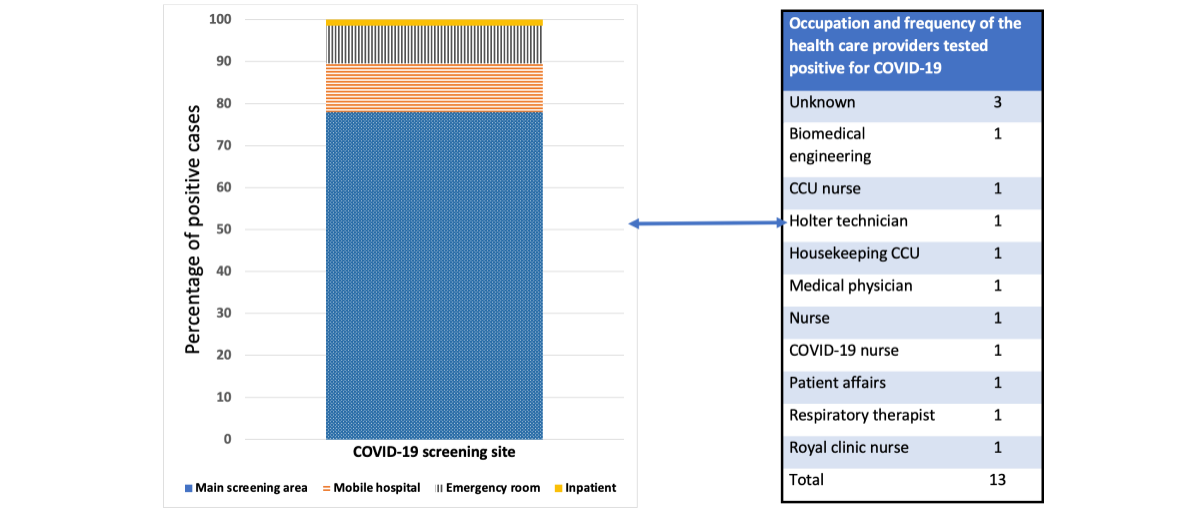
Percentages of screened cases as per the implemented policy and COVID-19 positivity among health care providers. COVID-19 nurse is a nurse allocated to the COVID-19 admission zone. CCU: critical care unit.

A screening area was identified to assess all potential COVID-19 cases, and a mobile hospital was established as a support facility to relieve pressure and ensure the smooth flow of patients. Primary health care providers, including doctors and nurses, attended to these areas; COVID-19 zones were allocated for positive cases admitted to the hospital based on the MOH recommendations with the intention of transferring them to MOH isolation facility, as these were the guidelines implemented during the first wave of COVID-19. The hospital policy steering committee also outlined other pathways to ensure that medical support for non–COVID-19 cases was adequate. Therefore, virtual clinics were established, elective admissions were minimized, and outreach services were created. The impact of the implemented policies and COVID-19 positivity among health care providers is presented in Figure 2. Notably, none of the primary health care providers assigned to the screening area or mobile hospital tested positive for COVID-19, suggesting the efficacy of the implemented policy.

### COVID-19 Exposure History and the Study Population Age

**Figure 3:**
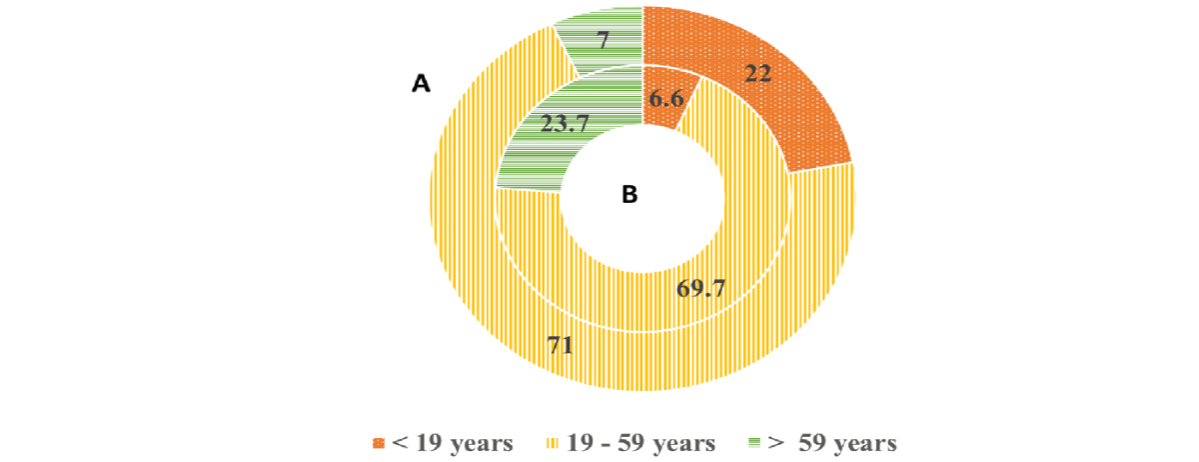
COVID-19 exposure history among the different age groups compared to international reports. (A) Our study population. (B) Fields et al's [4] study “with permission.” The main difference is the percentage of positivity cases among older adults (>59 years) suggestive of sociodemographic differences.

Notably, 7% (6/84) of the older adults in our study population had been exposed to a COVID-19–positive case; the percentages of exposure among the different age groups are presented in Figure 3.

### Predictors of Nontransfer to MOH Facility and Length of Hospital Stay

**Figure 4:**
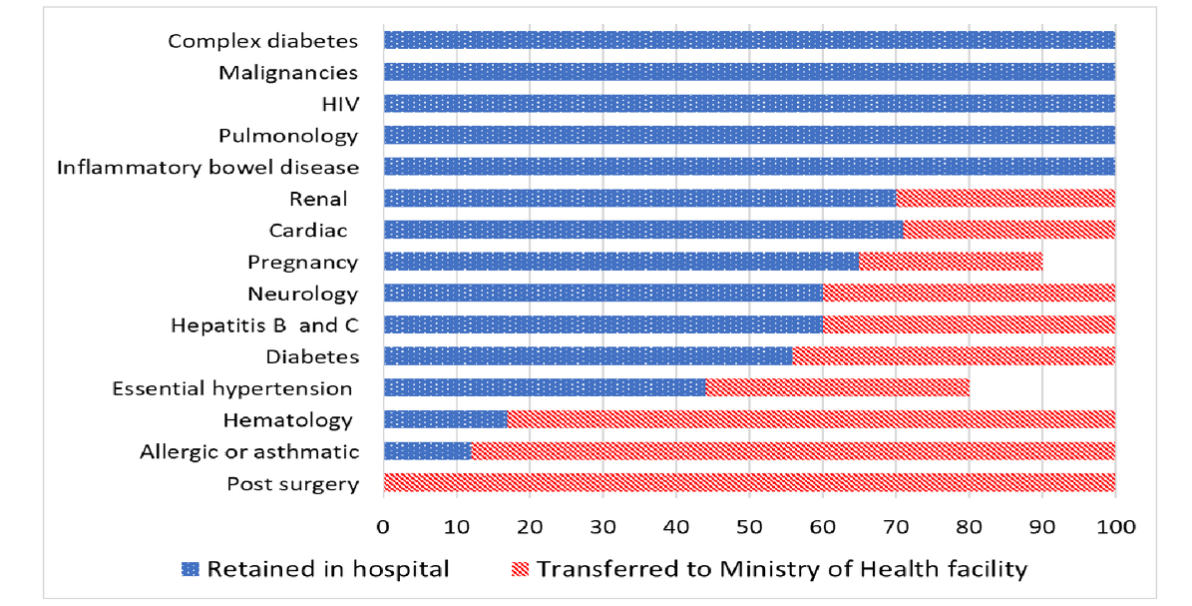
The impact of comorbidities on the transfer to Ministry of Health facility among individuals tested positive for COVID-19.

Data analysis revealed that the study population’s comorbidities had an impact on whether they would be transferred to the MOH facility (Figure 4). Notably, patients with significantly complex medical comorbidities were unlikely to be transferred to MOH isolation facilities.

COVID-19–positive cases with significant comorbidities requiring ongoing health care were retained in the hospital.

**Figure 5:**
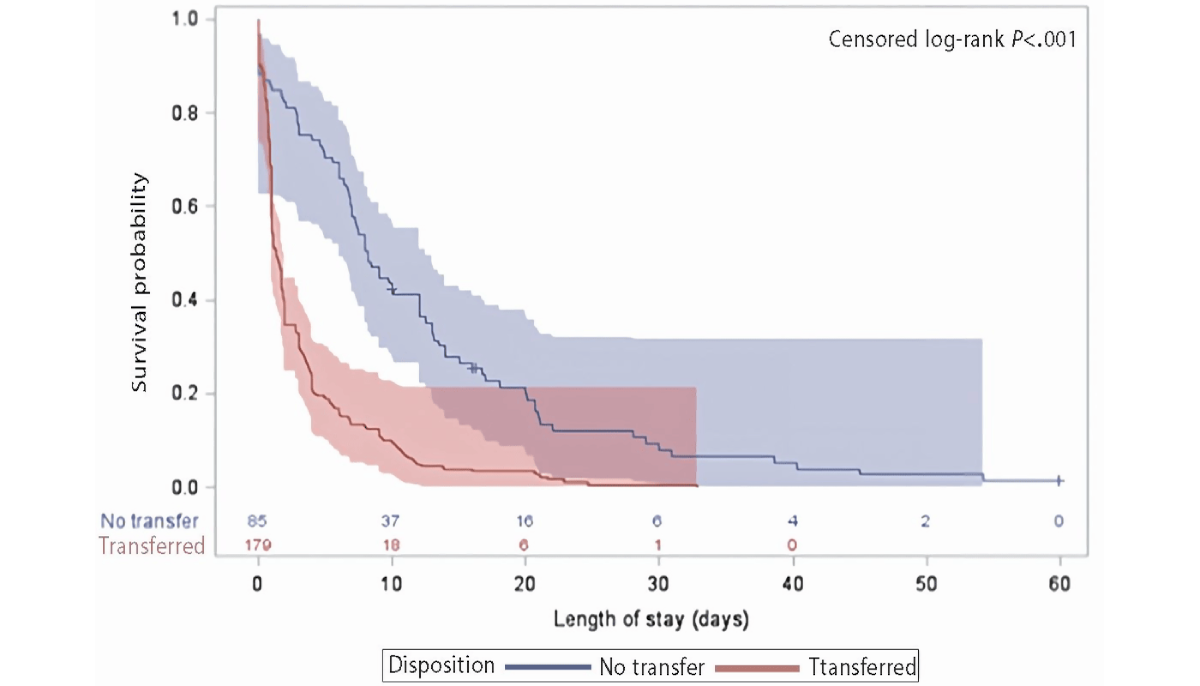
Time to transfer COVID-19–positive cases to the Ministry of Health facility. Product-limit survival estimates with number of subjects at risk and 95% Hall–Wellner bands.

The model revealed variability in the time taken for transfer to the MOH facility among COVID-19–positive cases (Figure 5). In the multivariate analyses, adjusted for potential confounders, age was a significant predictor of the length of hospital stay (3 days vs 12 days). The mean age was also statistically significantly and positively associated with the length of hospital stay, with older age being associated with a longer hospital stay (estimated coefficient 0.13; *P*<.001). This was similar to the need for ICU admission (estimated coefficient –1.82; *P*=.001), the need for lopinavir-ritonavir therapy (estimated coefficient 11.25; *P*<.001), symptoms suggestive of pneumonia (estimated coefficient 8.83; *P*=.005), and the need for blood cultures (estimated coefficient 10.67; *P*=.002*).* We did not assess the sensitivity of CURB-65 scores or COVID-19 visual scores in predicting the need for ICU admission, as 5% (14/275) of the study population needed ICU admission.

## Discussion

### Principal Findings

Our conceptual model revealed a low-risk population with a mean age of 42 (19.2) years, of which 19% (51/275) comprised older adults, and low CURB-65 scores were observed in 80% (220/275) of the study population. Furthermore, 53% (147/275) of the study population had no comorbidities. The laboratory results revealed mild leukopenia and lymphopenia; 20% (55/275) of the population showed thrombocytopenia, with an international normalized ratio within the reference range; 78% (214/275) of the study population had blood urea nitrogen levels <7mmo/L, which is in keeping with the low risk of mortality or ICU admission of 1.5% (4/275) and 5% (14/275), respectively. The low mortality rate of 1.5% was in keeping with what has been reported by the Saudi Arabian MOH [22], Alsofayan et al’s [23] multicenter study, and international research [24]. Additionally, the low mortality rate observed in our study might be attributed to the the demographic features identified via the conceptual model, which is in contrast to the 18% inhospital COVID-19 mortality reported by Hollar et al [25]. In their analysis, the age of 69 years, high Charlson comorbidity index, significant lymphopenia, neutrophilia, thrombocytopenia, elevated C-creative protein, lactate dehydrogenase, urea, and creatinine were identified as independent predictors of mortality. The impact of selectivity on ICU mortality was investigated by Filipe et al [26]; they evaluated COVID-19 inhospital mortality, ICU admission, and ICU-related mortality among the Sweden population; their analysis revealed a 15.1% inhospital mortality rate, 19.1% ICU admission rate, and a 23% ICU-related mortality rate. Analysis of the ICU-related mortality revealed that patients with a higher chance of survival were more likely to be admitted to the ICU; factors such as age <80 years and comorbidities other than dementia or liver disease were suggestive of an advanced medical care plan that refrained from intensive medical support in predefined patients. The overall ICU-related mortality rate in the study population was 4.7%.

Our analytical conceptual model revealed the efficacy of policies implemented to ensure health care providers’ safety; 4.7% (13/275) tested positive for COVID-19. The study also identified a conservative use of available resources, such as antibiotics and culture media; however, specific data regarding this finding were not reported. Based on our conceptual model, a high selectivity was implemented, which involved retaining patients with complex medical problems in the hospital; this approach did not increase the risk to attending health care providers nor did it increase mortality rates among patients. The low risk to attending staff was the result identified by the module.

A direct comparison of conceptual models is not always feasible, as it might address the same phenomena based on different attributes. Therefore, the results might be complementary but not similar. For instance, Demertzis et al [27] used a mathematical conceptual model to depict the successful downgrading of COVID-19 in Greece, while Snowodon’s [28] conceptual model forecasted the impact of informatics and technology in supporting key stakeholders at different stages of COVID-19 in the United States. However, the generalizability of their models is worth evaluating. Predictive modeling has been a key factor in mitigating the spread of COVID-19, as it has impacted the generation of medical guidelines and the implementation of policies. For instance, predictive models were developed to predict readiness to accommodate patients without COVID-19 in health care services [29]. Moreover, predictive models have captured the impact of medical knowledge gained over time on COVID-19 mortality. Snideret al’s predictive model [30] while Silk et al’s [31] social predictive model evaluated the impact of populations’ disease perception on adherence to NPI. They implemented a multiplex network, the outcome of which was “if the disease is perceived as high-risk with minimal reassurance, adherence to the NPI is ensured.” Burke et al [32] evaluated citizens’ vaccine uptake; their model revealed that trust and risk containment are key factors.

Additionally, the Jeddah tool and Salem were two predictive model tools developed by the Saudi MOH during the COVID-19 pandemic [22]. The former was used to guide the implementation or suspension of restrictions related to Umrah or Hajj, which are mass gathering religious activities, while the latter was used to guide the easing of lockdowns, NPIs, and social distancing measures.

### Limitations

The limitation of our conceptual model is that we could not evaluate the sensitivity of the COVID-19 visual score in predicting ICU admissions or morbidity. Unfortunately, the soft copies of the COVID-19 visual score were added to the hospital database, making manual extraction of the information impossible. A trained natural language processing system would have been helpful, but this was unavailable during the period of our study. The other limitation of our conceptual model is its simplicity. The intention of the simple model is to evaluate the current situation and aid in temporal guidelines, policy drafting, and implementation.

### Conclusions

With the tremendous development in information management systems and data repositories, data modeling might become a key factor in guiding health care policy and clinical research. Conceptual modeling, with its descriptive and analytical or predictive output, might be used to objectively allocate resources, optimize patient care, and guide policy generation implementation among health care services. Our conceptual model suggested that selectivity was implanted when deploying COVID-19–positive cases to the MOH isolation facilities. This implementation might have had a positive impact on the low mortality rate observed. The conceptual model revealed the efficacy of the policies implemented to protect health care providers. Further evaluation is needed to assess the impact of the conceptualization framework on health care policies.

## Abbreviations

CURB-65: confusion, urea, respiratory rate, blood pressure, and age >65 years
HbA_1c_: hemoglobin A_1c_
ICU: intensive care unit
MOH: Ministry of Health
NPI: nonpharmacological intervention
